# Prevention of the Vertical Transmission of HIV; A Recap of the Journey so Far

**DOI:** 10.3390/v15040849

**Published:** 2023-03-26

**Authors:** Maria Camila Cardenas, Sheila Farnan, Benjamin L. Hamel, Maria Camila Mejia Plazas, Elise Sintim-Aboagye, Dawn R. Littlefield, Supriya Behl, Sohan Punia, Elizabeth Ann L Enninga, Erica Johnson, Zelalem Temesgen, Regan Theiler, Clive M. Gray, Rana Chakraborty

**Affiliations:** 1 Pediatric Residency Program, Department of Pediatrics and Adolescent Medicine, Mayo Clinic, Rochester, MN 55905, USA; 2 Division of Pediatric Infectious Diseases, Department of Pediatric and Adolescent Medicine, Mayo Clinic, Rochester, MN 55905, USA; 3 Pediatric Residency Program, Nicklaus Children’s Hospital, 3100 SW 62nd Ave, Miami, FL 33155, USA; 4 Maternal Fetal Medicine Division, Department of Obstetrics and Gynecology, Mayo Clinic, Rochester, MN 33155, USA; 5 Department of Microbiology, Biochemistry, and Immunology, Morehouse School of Medicine, Atlanta, GA 30310, USA; 6 Department of Medicine, Division of Infectious Diseases, Mayo Clinic, Rochester, MN 55905, USA; 7 Division of Molecular Biology and Human Genetics, Biomedical Research Institute, Stellenbosch University, Francie van Zijl Drive, Tygerberg, Cape Town 7600, South Africa

**Keywords:** HIV, breastfeeding, prevention of vertical transmission, antiretroviral therapy, infants, prophylaxis

## Abstract

In 1989, one in four (25%) infants born to women living with HIV were infected; by the age of 2 years, there was 25% mortality among them due to HIV. These and other pieces of data prompted the development of interventions to offset vertical transmission, including the landmark Pediatric AIDS Clinical Trial Group Study (PACTG 076) in 1994. This study reported a 67.5% reduction in perinatal HIV transmission with prophylactic antenatal, intrapartum, and postnatal zidovudine. Numerous studies since then have provided compelling evidence to further optimize interventions, such that annual transmission rates of 0% are now reported by many health departments in the US and elimination has been validated in several countries around the world. Despite this success, the elimination of HIV’s vertical transmission on the global scale remains a work in progress, limited by socioeconomic factors such as the prohibitive cost of antiretrovirals. Here, we review some of the key trials underpinning the development of guidelines in the US as well as globally, and discuss the evidence through a historic lens.

## 1. Introduction

Globally, interventions to support the prevention of the vertical transmission (VT) of HIV have resulted in a precipitous decline in perinatal infections over the last three decades [1]. Fifteen countries have eliminated the vertical transmission of HIV, with Oman becoming the latest to achieve this goal [2]. These results are a far cry from the VT rates of 42% documented in the 1990s [3,4]; however, with the global VT rate being reported as 11.94% in 2021 [5], we still have far to go.

The interventions that have been effective in preventing VT for many thousands of children have not been feasible to implement in many settings. Factors contributing to this disparity include the prohibitive costs of ARVs; the risks as well as expenses associated with infant formula feeding; the limited availability of safe Cesarean sections for those in whom they are indicated; and investment in healthcare infrastructure. In this review, we will provide a brief overview of the pathophysiology of VT before discussing some of the interventions to reduce VT through a historical lens.

We recognize that another factor limiting progress towards the prevention of VT is ongoing stigma and discrimination against people living with HIV (PLHIV). We have therefore used non-stigmatizing language where possible. For example, we use “vertical transmission” instead of “mother-to-child transmission”. Additionally, we have tried to use language that is sensitive to transgender and gender-diverse populations, hence we use the term “pregnant people” instead of “pregnant women”. This may differ from the language used in the original research studies discussed.

## 2. Pathophysiology of Vertical Transmission

There are three opportunities for HIV to be transmitted from a pregnant person to an infant: during gestation, during delivery, and postnatally [6]. The risk of VT via all routes is highly influenced by the maternal viral load (VL) [7]. A fetus is susceptible to HIV infection while in utero as placental trophoblasts can become infected with the virus despite the absence of the CD4 receptor [7,8,9]. Based on data generated by our lab, we believe that maternal cytomegalovirus (CMV) viremia during coinfection can result in the inflammation of trophoblast cells, facilitating HIV migration into villous tissue and the upregulation of CCR5 in CD4+CCR5+ placental macrophages. This inflammatory cascade may promote intrauterine HIV transmission to the fetus [10,11,12]. Nevertheless, in utero transmission is the least effective route of VT, at a rate of about 5–10% in the absence of antiretroviral therapy (ART) [13,14]. The risk increases with an increasing maternal HIV viral load or with placental inflammation from coinfections (such as CMV) [7].

Intrapartum transmission is the most common route through which VT occurs, accounting for 15–20% without ART [13,14]. VT is facilitated by infected maternal blood and secretions from the birth canal coming into contact with the mucosal surfaces of the neonate [7]. A prolonged duration of a membrane rupture increases the rate of VT [15,16], thus minimizing the rupturing of membranes and, in some cases, performing an elective Cesarean section reduces infant exposure and can decrease the risk of VT [17].

**Figure 1:**
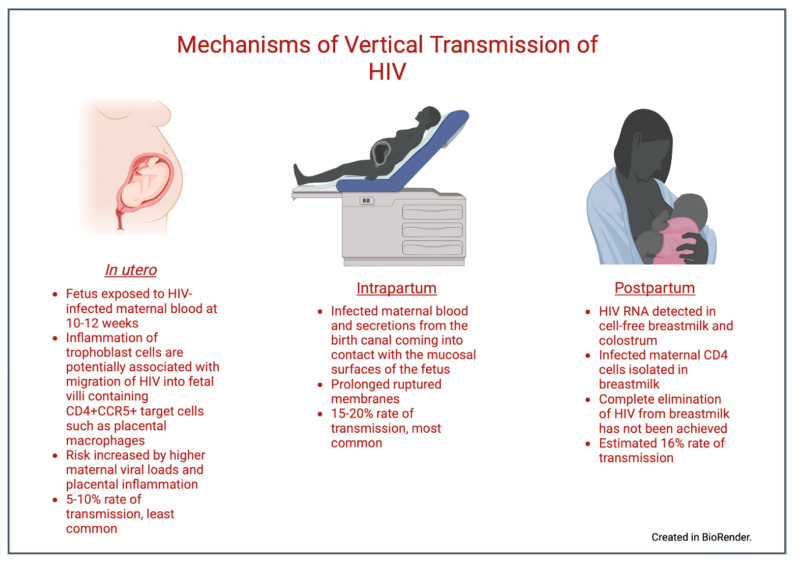
Mechanisms of vertical transmission. Created in BioRender.

Postnatal transmission usually occurs through breastfeeding. Maternal CD4 cells associated with an actively replicating or quiescent virus have been detected in breast milk [18], and HIV RNA has even been detected in cell-free breast milk [19,20]. There has been no trial that has been successful in completely eliminating HIV from breast milk [18], and the VT rate via this route is estimated to be 16% [21]. Therefore, almost all industrialized countries have historically recommended the complete avoidance of breastfeeding when safe water and formula are readily accessible [22,23]. Figure 1 summarizes the three routes of VT.

## 3. Neonatal HIV Infection

Case reports in the early 1980s first described infants born to people with AIDS showing signs and symptoms of a similar immunodeficiency [24,25]. HIV in a neonate can present with a critically unwell infant who dies early in life. In the pre-ART era, rapid disease progression and death by 24–36 months was documented by a number of investigators in the United States (U.S.) and Malawi [26,27]. Children infected in the perinatal period may remain asymptomatic beyond infancy [28,29]. Manifestations in childhood include faltering longitudinal growth, developmental delay, lymphadenopathy, pneumonitis, nephropathy, cardiomyopathy, diarrhea, candidiasis, hepatitis, neoplasia, recurrent infections, and death [28]. In the sections below we will highlight some of the landmark studies conducted over the last 30 years and discuss how their findings have influenced the development of guidelines and strategies aimed at reducing VT.

## 4. HIV Testing

The World Health Organization (WHO) has developed a system of five Cs in regard to HIV testing: consent, confidentiality, counseling, correct test results, and connections to care [30]. These principles are critical for all countries, but especially for those that lack robust healthcare infrastructures. The WHO recommends HIV testing in high-burden areas and in individuals with clinical presentations that may reflect an underlying immunodeficiency, such as malnutrition or a sexually transmitted infection (STI) [30].

### 4.1. Pre-Pregnancy

In the US, approximately 34% of infants with perinatal HIV exposure are born to people who do not know that they had acquired HIV prior to their pregnancy [1]. Hence, it is imperative that providers counsel all people of child-bearing potential on the importance of HIV testing and pre-exposure prophylaxis (PrEP) to minimize VT. In the US, approximately 50% of all pregnancies are unplanned, with this number being closer to 70% in people living with HIV [31,32,33,34]. Individuals of child-bearing age living with HIV should receive evidence-based counseling on appropriate contraception methods if they are seeking to avoid pregnancy, since some antiretrovirals (ARVs), such as ritonavir, reduce the efficacy of hormonal contraceptives [35,36,37]. PLHIV who desire to become pregnant should aim to maintain viral suppression with ART prior to conception, during pregnancy, and postnatally.

### 4.2. During Pregnancy

The Pediatric AIDS Clinical Trial Group Study (PACTG076) was a randomized controlled trial investigating the role of ARVs in the prevention of VT [38]. This landmark study led to a paradigm shift in the management of HIV in pregnancy. One of the first recommendations implemented based on PACTG076 was expansion of HIV testing in pregnancy—from the selective HIV testing of “high-risk” groups to voluntary routine testing for all pregnant women in US [39,40]. These recommendations were further modified by the American Academy of Pediatrics (AAP) and the American College of Obstetrics and Gynecology (ACOG) for universal “opt-out” testing [41]. In the US, the universal testing of all pregnant people is recommended in the first trimester, with repeat testing in the third trimester mandated in some areas [22,42]. In contrast, South Africa strongly advises HIV testing for all pregnant people every 3 months throughout pregnancy and during labor [43]. This difference reflects the much higher burden of HIV infection in South Africa. Despite guidelines for universal screening in pregnancy, <80% of pregnant people in the US report receiving an HIV test [44,45], with those without health insurance at a greater risk of not being tested [44]. A number of other studies in the US note that the rate of re-testing in the third trimester is well below the target level [46,47,48], with one study showing only 28% of eligible people received a third-trimester test [46].

In pregnant PLHIV, the viral load (VL) is directly linked to the likelihood of VT [49]. Viral suppression is the goal of all HIV-related interventions during pregnancy, and regular VL testing throughout pregnancy is recommended, particularly at 36 weeks (or four weeks prior to anticipated delivery), as this will help inform decisions regarding the mode of delivery [22,30,50]. Viral rebound can even occur in cases of good adherence to ART. This risk increases with cocaine use and hepatitis C [51]. The close monitoring of VL is essential in all pregnant PLHIV but is particularly important in the presence of these and other known risk factors.

### 4.3. In Labor

In the US, pregnant people of an unknown HIV status, including those from “at-risk” groups who test negative in the first trimester and are not re-tested in the third trimester, should undergo expedited antigen/antibody HIV testing in labor. While the WHO guidelines do not have any guidelines for HIV testing in labor specifically, several countries have recommended the practice in their individual guidelines [37,43,52].

### 4.4. Postpartum

If not tested before or during labor, rapid testing in the immediate postpartum period is important for the health of a postpartum person and their infant. If this or any other pre-labor test is positive for HIV then the neonate should be tested. Some jurisdictions mandate the HIV testing of newborns; New York, for example, requires the HIV testing of all newborn infants [53]. Maternal ART can impact the results of commonly administered HIV tests [54,55], and providers should ensure that they are using the most appropriate test for this age group. Testing should be repeated in exposed infants even if results were negative at birth, with most guidelines recommending a risk stratification approach to decide on the frequency of testing [3,22,52].

## 5. Pre-Exposure Prophylaxis (PrEP)

Susceptibility to HIV acquisition is greater during the periconception period through to pregnancy until approximately six months postpartum, likely due to repeated sexual intercourse without barrier methods; increased innate and suppressed adaptive immunity; inflammation of the genital tract; reduced integrity of the vaginal epithelium; and alterations in the genital microbiome [56,57,58,59]. HIV incidence among pregnant people is two to six times higher than that of non-pregnant people [60,61,62,63,64]. Additionally, the risk of VT is higher with recent HIV acquisition, reflecting a high VL during acute seroconversion [65,66].

PrEP has been shown to be effective at reducing the horizontal transmission of HIV [67] and is currently recommended to reduce the rate of new infections in groups that are at risk of pregnancy and HIV acquisition [22]. A 2016 meta-analysis suggested that the real-life use of PrEP (with an estimated adherence of 75%) decreases HIV acquisition by 61% (relative risk: 0.39; 95% CI: 0.25–0.50) [68]. These results are encouraging, particularly as studies across the US, Kenya, and South Africa have documented that adherence to PrEP during the periconception period is generally much higher than the estimated 75% [69,70,71]. As a result, international guidelines recommend that PrEP is commenced for any individual at risk of HIV acquisition and should not be discontinued when a person becomes pregnant [30].

## 6. Antiretroviral Therapy (ART)

In the sections below we will discuss the usage of ART to reduce VT in the pre-pregnancy, antenatal, delivery and postpartum phases.

### 6.1. Pre-Pregnancy

All PLHIV are advised to aim for sustained viral suppression (a VL of < 50 copies/mL) before attempting conception for their own health and to reduce the risk of VT [22]. All people who are diagnosed with HIV for the first time whilst attempting to conceive should start ART immediately [22,72] Pregnant PLHIV who are established on effective ART should continue their regimen, provided that it is safe and effective in pregnancy. Non-standard regimens may have altered pharmacokinetics in pregnancy, which may necessitate a change [73]. If possible, any changes to a treatment regimen should be made (and efficacy as well as safety demonstrated) prior to conception, as stopping ART during pregnancy, even for a short time, can lead to an increase in the plasma VL and an increase in the risk of VT [74].

### 6.2. During Pregnancy and Peripartum

It is recommended worldwide that all PPHIV receive ART throughout their pregnancy, delivery, and postpartum [30]. ART toxicity can occur in pregnancy, and pregnancy symptoms, such as severe nausea and vomiting, can influence the tolerability of various ART regimens. If an ARV regimen needs to be discontinued during pregnancy, the recommendation is that all ARVs are stopped and an alternative regimen commenced with as short a delay as possible [22].

Before the implementation of widespread antepartum ART, the rate of VT was approximately 25% [69], rising to 40% with breastfeeding [3,4]. In 1994, the landmark PACTG076 trial was published—this double-blind RCT investigated the role of antenatal zidovudine (ZDV) in HIV-affected pregnancies and documented a dramatic 67.5% relative risk reduction in vertical HIV transmission (*p* = 0.00006), with 8.3% of the children born in the intervention arm testing positive for HIV infection at 18 months of age, compared to 25.5% in the placebo group [38]. This study was one of the first demonstrations of treatment as prevention in medicine, and, based on the conclusions, the use of antenatal as well as intrapartum ZDV was implemented widely; however, the intravenous ZDV regimens were difficult to implement in many settings, which limited uptake. Less intensive ZDV regimens were investigated by groups in Côte d’Ivoire and Burkina Faso, where investigators documented that shorter courses led to reduced VT compared to the baseline, but with less pronounced results than those observed with the more intensive PACTG076 regimen [75,76].

Sperling et al. hypothesized that one of the mechanisms by which ZDV reduced VT in these trials was protection from HIV during the process of delivery [77]. With this, in addition to keeping the difficulties in administering intensive ZDV regimens in mind, investigators in Uganda evaluated the safety as well as efficacy of short-course oral nevirapine (NVP) in the HIVNET 012 trial. This double-blind RCT randomized PPHIV to receive either oral ZDV or oral NVP intrapartum [4]. Those in the NVP arm received a single dose of oral NVP at the onset of labor, and their infants received a single dose of oral NVP within 72 h of birth. Pregnant people in the ZDV arm received oral ZDV at the onset of labor followed by subsequent doses during labor, and their infants received oral ZDV for seven days [4]. The initial results showed a 47% reduction in VT in the NVP compared to the ZDV arm [4]. The results of a prolonged follow-up showed that the NVP arm continued to have significantly lower rates of HIV infection at 18 months [78,79]. These data demonstrated that a short course of NVP could provide a benefit in resource-limited settings and bought tangible optimism to patients and providers alike, in addition to a commitment from Boehringer Ingelheim to provide NVP at little or no cost to most low–middle-income countries (LMICs).

These breakthroughs led to examining what other factors influence VT through the Bangkok Collaborative Perinatal HIV Transmission Study, the PACTG, and the Women and Infants Transmission Group Study (WITS) in the US. In the Bangkok study, conducted from 1992 to 1994, 68 of 281 infants born to PPHIV acquired HIV (a transmission rate of 24.2%). Birthing parents of infected infants had a significantly higher median plasma HIV RNA VL around the time of delivery than birthing parents of those who remained uninfected with HIV. No transmission occurred with a maternal VL of< 2000 copies/mL. Two-thirds of transmission events that occurred were attributed to a maternal VL of >10,000 copies/mL [80]. Over the same time period, the PACTG examined 480 pregnancies where ZDV was administered in line with best practice at the time. Again, it was demonstrated that the VT rate increased with an increasing maternal VL and that no VT occurred with a maternal VL of <1000. Multivariate analyses in these studies showed that prematurity, vaginal delivery, a low birth weight, and the length of a membrane rupture were independently associated with VT [80]. The maternal VL is the best predictor of the risk of the perinatal transmission of HIV. Lowering the maternal VL with ART is the most important mechanism by which VT can be prevented [81].

The roll-out of ART became the standard of care in subsequent years throughout many countries. The PROMISE trial, published in 2016 [82], was conducted across seven countries and investigated the rate of VT with three different ARV regimens: the rate of VT was significantly lower in participants who received combination ART (0.5%) compared to ZDV monotherapy (1.8%), but the rate of maternal and neonatal adverse events was significantly higher in those receiving ART than with ZDV alone. The overall conclusions from PROMISE and other studies on reducing the VT of HIV are that the benefits of ART in pregnancy outweigh the risks of this intervention.

The Botswana Harvard AIDS Institute Partnership conducted birth outcome surveillance in Botswana to evaluate the prevalence of birth defects associated with antenatal exposure to ARVs. The investigators noted a potential early signal for neural tube defects (NTDs) associated with periconception exposure to the integrase inhibitor dolutegravir (DTG) [83]. This led to the removal of DTG from the list of preferred medications on many guidelines. Continued observation in the same study documented a decline in the difference between the incidence of NTD in DTG-exposed pregnancies and DTG-unexposed pregnancies. A 2021 update documented the difference in the prevalence of NTDs between DTG-exposed and DTG-unexposed infants as being 0.05%, which was not statistically significant. Based on these updated data, DTG was reintroduced into the WHO guidelines as a preferred ARV throughout pregnancy [30].

The significant improvement in HIV-related morbidity and mortality in individuals who were able to maintain good adherence to ART was reflected in a persistently undetectable VL. This prompted questions on the necessity of certain interventions in PPHIV that had become the standard of care in the 1990s; in particular, the requirement of intrapartum ZDV. Briand and colleagues evaluated the impact of intrapartum ZDV according to VL and obstetrical conditions in the deliveries of 11,538 pregnant PLHIV from the French Perinatal Cohort. In this study, the rate of VT in pregnancies with a VL of <400 copies/mL of 0% (0/341) when intravenous intrapartum ZDV was not given, compared to a rate of 0.6% (42/7576) in similar patients who did receive intravenous intrapartum ZDV. This difference was not statistically significant (*p* = 0.17). The authors concluded what is now reflected in many guidelines: that for people with a low VL at delivery, intravenous ZDV is not necessary [84].

**Figure 2:**
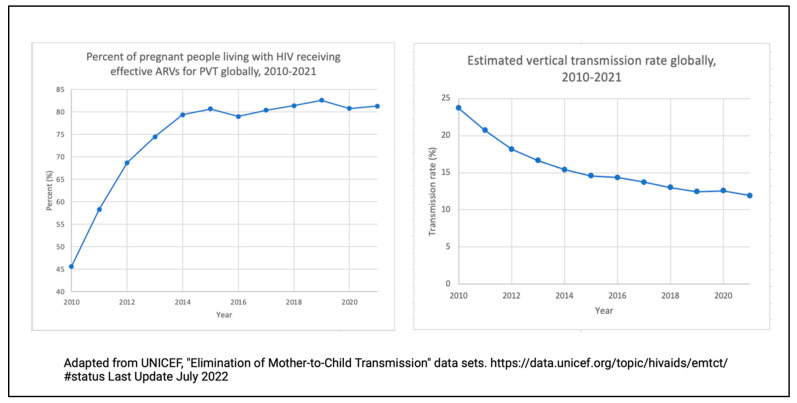
Line graphs illustrating the percentage of HIV-positive people on antiretroviral medication in terms of the prevention of VT from 2010 to 2021 (**left**) and the estimated vertical transmission rate globally from 2010–2021 (**right**).

Lowering the maternal VL with ART is the most influential method in reducing VT, as illustrated in Figure 2.

## 7. Mode of Delivery

Prior to the widespread use of ART to suppress the VL, the European Mode of Delivery Collaboration study randomized PPHIV to elective Cesarean sections at 38 weeks or normal spontaneous vaginal deliveries. Both groups were studied in the absence and presence of ZDV. VT rates ranged from 20% in the arm with no interventions (no Cesarean section and no ZDV) to 1% with both (Cesarean sections and ZDV). Transmission rates for Cesarean sections without the administration of ZDV were reported to be 4%, demonstrating the efficacy of operative delivery as an intervention to reduce VT [4].

With the widespread administration of suppressive ART, Briand and colleagues analyzed data from the French Perinatal Cohort and >4000 pregnant PLHIV for mode of delivery practices. In their article from 2013, the VT rate did not differ according to mode of delivery in term pregnancies when the maternal VL was beneath the level of detection. The authors concluded that, in the absence of other indications for operative delivery, PPHIV who are adherent to ART and maintain VL suppression can deliver vaginally with a minimal risk of VT. Vaginal delivery was also associated with lower rates of maternal infection postpartum (0.02%) compared to those who delivered via Cesarean sections (0.04%) [85].

South African and Indian guidelines only recommend Cesarean sections for obstetric indications and do not recommend this intervention to reduce VT [43,50]. Many other countries, including the US, the United Kingdom (UK), Nigeria, and Kenya recommend that, if the VL is unknown or high within four weeks of delivery, Cesarean sections should be scheduled at 38 weeks of gestation [22,37,72,86]. If a Cesarean section is indicated for other reasons, the intervention should be performed according to standard obstetric protocols.

In 1996, the WITS study followed a cohort of 525 pregnant PLHIV and reported increased VT when the duration of ruptures of membranes exceeded 4 h [15]. In addition, a 2001 meta-analysis of 15 prospective cohort studies from North America and Europe demonstrated that the risk of VT increased by 2% for each hour between a membrane rupturing and delivery, including with the administration of antepartum, peripartum, and postnatal ZDV [16]. Neither the WITS study nor the 2001 meta-analysis factored in HIV viral suppression with ART, which was not the standard of care when the original research was conducted. Since 2000, a number of studies have demonstrated that, in cases where there is adequate viral suppression, there is no increased risk of VT associated with an increased length of time between a membrane rupturing and delivery, up to 24 h [87,88,89,90]. There is a paucity of evidence on this topic with very prolonged ruptures of membranes (>24 h); therefore, guidelines suggest individualized management [22]. Avoiding a prolonged membrane rupture has been adopted into the guidelines of several countries, including India and Kenya [37,50].

Other considerations for vaginal delivery include instrumental delivery, spontaneous premature labor, and water births. Before data from the era of ART were available, HIV was considered a relative contraindication to instrumental vaginal delivery with forceps or a vacuum device. A review in the UK of 9072 deliveries between 2008 and 2016 concluded that instrumental delivery is associated with a minimal risk of VT among PPHIV with a low VL [91]. Based on these data, it is generally recommended that instrumental delivery follows standard obstetric indications in patients with an undetectable VL. This intervention should be avoided if the VL is >/= 50 copies/mL [22]. One mechanism by which HIV is transmitted to an infant during delivery is through damaged vaginal epithelia [92], which raises questions about the risk associated with vaginal tears or episiotomy. No high-quality data exist to address these questions in the ART era, and most guidelines suggest that indications for episiotomy in the context of HIV should not differ from standard obstetric indications [22]. Outcomes for premature babies are greatly improved through the administration of antenatal steroids [93], which should be provided to HIV-exposed infants. There remains some degree of skepticism of water births, although a systematic review and meta-analysis in people without HIV indicated that they are not associated with worse outcomes for neonates [94]. There is scant safety data regarding water births in PPHIV. Water births are not mentioned in the US guidelines; however, the British HIV Association (BHIVA) guidelines do state that PPHIV who have a VL of < 50 copies/mL should be supported if desirous of a water birth, in line with guidance for pregnancies without HIV [22,72].

## 8. Other Considerations in the Care of Pregnant PLHIV

From pre-conception care to the postpartum period, a multidisciplinary approach is strongly recommended [22,30,72]. A study in Texas demonstrated that HIV-adapted group prenatal care was associated with a high retention in the clinic and a trend towards a lower maternal VL [95]. These holistic interventions can be difficult to implement, and the best approach is likely to be one that is complementary to the community in which it exists. The PURE study in Malawi is a randomized trial investigating the rates of VT between groups who had peer support in the community versus in a clinic setting [96].

Screening for infectious and non-infectious morbidities is important in the care of pregnant people. For some pregnancy-related screening protocols the HIV status of the person alters the recommendations, including early glucose screening for pregnant people receiving protease-inhibitor-based ART [97]. In the circumstance that a PPHIV has indications for invasive testing, such as amniocentesis, this should be deferred until viral suppression is achieved [98,99].

Delayed cord clamping, the practice of leaving the umbilical cord intact (the ACOG recommends 30–60 s) after the delivery of a baby, is recommended for most deliveries in the non-HIV setting [100,101,102]. This intervention has not been studied in PPHIV with a high VL. No specific recommendation is made in the US or UK guidelines; however, both Nigeria and India have integrated this practice into their respective national HIV guidelines [50,86].

Providers should be mindful of drug–drug interactions for PPHIV, particularly during the management of delivery, as these may impact management decisions. For example, some protease inhibitors will alter the metabolism of methergine, which is commonly used for managing postpartum hemorrhage [103].

## 9. Postpartum Interventions

### 9.1. Antiretroviral Medication for the Birthing Parent

The Mma Bana trial was conducted in Botswana between 2006 and 2008: PPHIV with a CD4+ T cell count of >200 cells/mm^3^ and no AIDS-defining illness were randomized to receive either nucleoside reverse transcriptase inhibitor (NRTI)-based ART or protease inhibitor (PI)-based ART during the postpartum period. Breastfeeding was not excluded. A low level of postnatal VT (1.1%) was detected across all participants, associated with a maternal VL of <400 copies/mL [83]. Both maternal and infant mortality rates decreased at 24 months to 1.9% and 5.2%, respectively [104]. In line with national guidelines at the time, maternal ART was discontinued after weaning for participants with CD4+ T cell counts of >200 cells/mm^3^ and no AIDS-defining illness. An increase in the mortality rate occurred once maternal ART was discontinued, prompting the argument that ART should be continued indefinitely in PLHIV In 2013, based on a growing body of evidence [18,79,105,106,107,108,109,110], the WHO shifted towards “Option B+”, an approach in which all PLHIV who are pregnant or breastfeeding initiate lifelong ART independent of CD4+ count. Ninety-five percent of Global Plan priority countries, where 90% of PPHIV reside, endorsed Option B+, and the percentage of pregnant PLHIV receiving ART increased from 36% in 2009 to 77% in 2015, which led to a reduction in the number of new pediatric HIV infections in these countries by 60% [111].

It is now a global recommendation that all PLHIV remain on ART indefinitely [30,72]. Providers should bear in mind that the postpartum period presents significant challenges to ART adherence; there is evidence that case management to help patients navigate the complexities of healthcare systems can increase retention in care and lead to better rates of viral suppression [112]. Additional counseling for adherence to ART in both parents and infants is strongly recommended by the WHO [30].

### 9.2. Antiretroviral Medication for Infants

In 1998, Wade and colleagues published a retrospective study on mother–infant dyads who had received an abbreviated ZDV regimen. This study reviewed data from the HIV PCR testing service of the New York State Department of Health and evaluated the effects of an abbreviated regimen with a specific focus on the role of the timing of ZDV administration. The findings of this study drew attention to the importance of administering postnatal ZDV as soon as possible after birth. When prophylaxis was administered within the first 48 h of life the rate of VT was 9.3%; when the initiation of ZDV was delayed until day 3 of life or later the rate was 18.4% [113].

In 2012, the HIV Prevention Trial Network 040 study reported on the safety and efficacy of adding ARVs postpartum in addition to standard ZDV prophylaxis in infants born to PPHIV with a high VL near the time of delivery. Across the Americas and South Africa, 1684 HIV-exposed infants received one of three postnatal regimens, either as a one-drug (ZDV), two-drug, or three-drug regimen. The investigators concluded that in neonates whose birthing parents were not virologically suppressed during pregnancy, the postnatal administration of the two- or three-drug ARV regimen was superior to ZDV alone for the prevention of VT [114].

**Figure 3:**
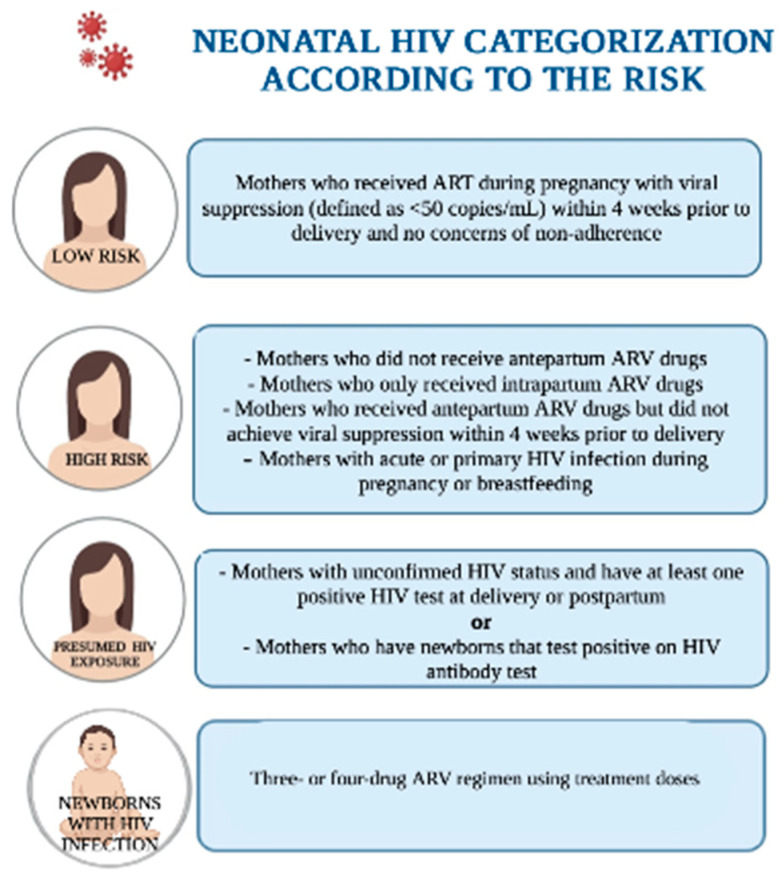
“Neonatal HIV Categorization According to the Risk”. Adapted from Recommendations for the Use of Antiretroviral Drugs During Pregnancy and Interventions to Reduce HIV Transmission in the United States. 30 December 2021. Created in BioRender.com.

Currently, the Swiss Federal Office of Public Health does not recommend the administration of any ARV to neonates born to PLHIV who demonstrated consistent ART use during pregnancy and had sustained viral suppression [115]; however, the best practice in all other countries is that all newborns who have been exposed to HIV should receive ART to reduce the risk of VT [22,30,37,43,50,72,86]. Most countries recommend a risk-stratified approach to these decisions. The recommendations from Health and Human Services in the US [22] are schematically represented in Figure 3.

## 10. Infant Feeding

Breastfeeding decreases all-cause morbidity and mortality from gastroenteritis, pneumonia, and malnutrition in children under 5 years of age when given exclusively for the first 6 months of life [116,117]. Breastfeeding reduces the incidence of respiratory and gastrointestinal infections, asthma, celiac disease, inflammatory bowel disease, obesity, diabetes, and childhood leukemia. Breastfeeding is also associated with a reduction in maternal breast and ovarian cancer, as well as postpartum depression [117,118,119,120,121,122,123]. These benefits must, however, be balanced against a risk of VT, as transmission may still occur through breast milk even with an undetectable maternal plasma VL [18]. The AAP and guidelines from all industrialized countries (with the exception of Switzerland) advise that HIV is a contraindication to breastfeeding and recommend full formula feeding for children born to PLHIV provided that there is a safe alternative available [115,124]. BHIVA guidelines stipulate that any PLHIV who has been advised not to breastfeed should be provided with an adequate supply of free formula for their infant [72]. This expensive intervention is not implementable worldwide, and in settings where infant formula is neither safe, affordable, nor readily available, breastfeeding is essential to an infant’s survival. The WHO and United Nations Children’s Fund (UNICEF) recommend that PLHIV in areas with a high prevalence of mortality from diarrhea, pneumonia, and malnutrition should exclusively breastfeed their infant whilst taking ART to minimize the risk of VT [52]. This recommendation is echoed in country-specific guidelines from Kenya [37], South Africa [43], India [50], and Nigeria [86].

The 2012 BAN (breastfeeding, antiretroviral, and nutrition) study in Malawi compared maternal and infant interventions during breastfeeding. In the study, 2369 breast-feeding PLHIV with a CD4+ T cell count of at least 250 cells/mm^3^, as well as their infants, were randomly assigned to receive a maternal ARV regimen; infant NVP; or no extended postnatal ARV regimen (control group). All PLHIV and infants with HIV exposure received perinatal prophylaxis with single-dose NVP and 1 week of ZDV plus lamivudine. The study found that the use of either a maternal ARV regimen or infant NVP for 28 weeks was safe and effective in reducing VT during breastfeeding [125]. Serious adverse events associated with the administration of ART by breastfeeding mothers were uncommon in infants. In two studies that compared the efficacy of maternal ART to infant NVP prophylaxis without maternal ART during breastfeeding, there were no significant differences in adverse events observed between the study arms [109,116]. Of the drugs that have been studied, which include NNRTIs, PIs, and NRTI, the concentrations in breast milk were very low [126,127,128], with little to no drug detected in the infants’ blood. Infants exposed to these subtherapeutic drug levels may be at an increased risk of ARV resistance, which may be problematic with HIV acquisition [129,130].

Several studies have shown that, in environments where safe infant formula is not available, exclusive breastfeeding reduces the risk of VT and increases HIV-free survival compared to mixed feeding [131,132,133,134,135,136,137,138]. Mixed feeding, that is, feeding an infant a combination of breast milk and formula milk, is discouraged, with higher rates of VT noted compared to either exclusively breast milk or exclusively formula. This may be due to the disruption of the intestinal epithelium and a pro-inflammatory milieu increasing the risk of HIV entry into target cells [136].

The latest iterations of the guidelines in some high-income countries, including the UK [23], the US [12], and Switzerland [115], include sections with recommendations for PLHIV who desire to breastfeed. Postpartum PLHIV who desire to breastfeed should be virally suppressed, reflecting optimal adherence to ART. Recommendations include patient-centered and evidence-based counseling on infant feeding options. If PLHIV opt to breastfeed after counseling, recommendations include providing support in risk-reduction measures to minimize VT, including the avoidance of mixed feeding [22,23,115]. Advice should be given to any HIV-positive person who is breastfeeding to avoid the abrupt cessation of breastfeeding, as there is evidence to suggest that rapid weaning may be associated with poor outcomes in infants [138].

Premastication, the practice of prechewing food before administering the food to another individual for their consumption, is practiced around the world, often during the weaning period. Data from the US from the Infant Feeding Practices survey conducted in 2009 reported that 10.5% of respondents admitted to premasticating food for their infant [139]. A similar survey in South Africa showed that 69% of the respondents practiced premastication for children [140]. HIV has not been detected in saliva but has been detected from bleeding gingivae, which may result in viral transmission. It is important that healthcare providers routinely inquire about this practice and counsel caregivers on safer feeding options [22].

Conflicting advice and perceptions about infant feeding practices can contribute to psychological stress in PLHIV, which can impact mental and physical health [132]. In many cultures there are social and societal expectations encouraging exclusively breastfeeding over formula feeding. As a result, many parents experience considerable stigma if they choose to avoid breastfeeding. Providers must be cognizant of a patient’s perceptions around infant feeding and the various influences that inform them [116,117].

## 11. Routine Postnatal Care

Children living with HIV and, oftentimes, children who are HIV-exposed but uninfected have worse health outcomes compared to HIV-unexposed and uninfected children [141]. Immunization is critically important for these children. Recommendations worldwide call for multidisciplinary care, which should include evidence-based advice on family planning, case management, and mental health.

## 12. Conclusions and Future Directions

**Figure 4:**
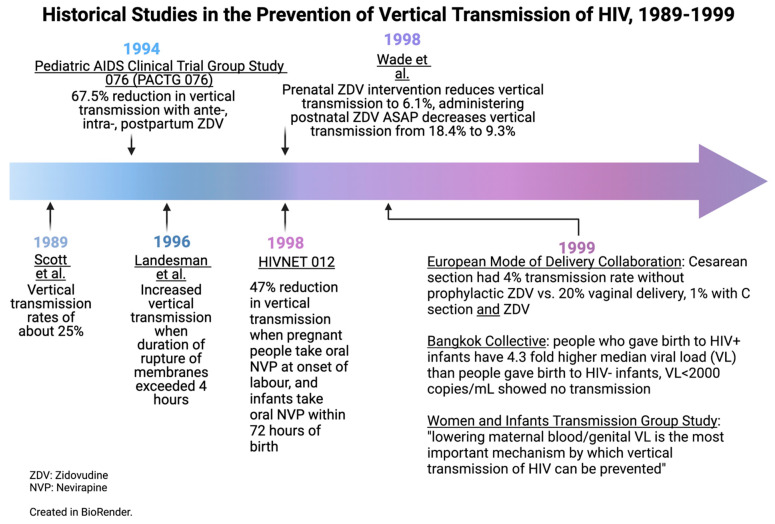
Historical studies in the prevention of vertical transmission of HIV, 1989–1999. Created in BioRender.com.

**Figure 5:**
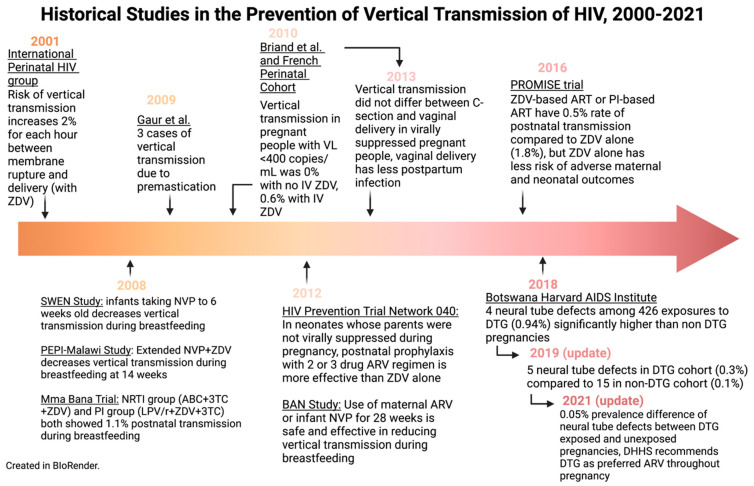
Historical studies in the prevention of vertical transmission of HIV, 2000–2021. Created in BioRender.com.

In this review, we have discussed some of the interventions which have led to dramatic reductions in VT rates over recent decades. Figure 4 and Figure 5 outline the historical studies discussed in this paper; however, ongoing challenges in infrastructure, health economics, and misunderstandings surrounding HIV may limit progress towards the goal of global elimination. Future directions for the accomplishment of the total elimination of the vertical transmission of HIV will move toward the increased affordability of ARVs, more research around ARV resistance, and more efforts to destigmatize HIV/AIDS around the world. Nevertheless, the picture of HIV’s vertical transmission today is extremely encouraging compared to that of the late 1990s. Given that several countries have eliminated VT, there is real hope of an HIV-free generation in the future.

## Data Availability

Not applicable.
